# Flight activity and effort of breeding pied flycatchers in the wild, revealed with accelerometers and machine learning

**DOI:** 10.1242/jeb.247606

**Published:** 2024-10-10

**Authors:** Hui Yu, Shujie Liang, Florian T. Muijres, Jan Severin te Lindert, Henrik J. de Knegt, Anders Hedenström, Koosje P. Lamers, Per Henningsson

**Affiliations:** ^1^ Experimental Zoology Group, Wageningen University, 6708 WD Wageningen, The Netherlands; ^2^ Shan Shui Conservation Center, Beijing, China; ^3^ Wildlife Ecology and Conservation Group, Wageningen University, 6708 WD Wageningen, The Netherlands; ^4^ Department of Biology, Lund University, Naturvetarvägen 6A, 223 62 Lund, Sweden; ^5^ Conservation Ecology Group, Groningen Institute for Evolutionary Life Sciences (GELIFES), University of Groningen, 9700 CC Groningen, The Netherlands; ^6^ Danish Hydraulic Institute (DHI), Agern Alle 5, 2970 Hørsholm, Denmark

**Keywords:** Parental care, Foraging, Morphology, Wing loading, Forest habitat, VeDBA

## Abstract

Flight behaviours of birds have been extensively studied from different angles such as their kinematics, aerodynamics and, more generally, their migration patterns. Nevertheless, much is still unknown about the daily foraging flight activity and behaviour of breeding birds, and potential differences among males and females. The recent development of miniaturized accelerometers allows us a glimpse into the daily life of a songbird. Here, we tagged 13 male and 13 female pied flycatchers (*Ficedula hypoleuca*) with accelerometers and used machine learning approaches to analyse their flight activity and effort during the chick rearing period. We found that during 2 h of foraging, chick-rearing pied flycatchers were flying on average 13.7% of the time. Almost all flights (>99%) were short flights lasting less than 10 s. Flight activity changed throughout the day and was highest in the morning and lowest in the early afternoon. Male pied flycatchers had lower wing loading than females, and in-flight accelerations were inversely correlated with wing loading. Despite this, we found no significant differences in flight duration and intensity between sexes. This suggests that males possess a higher potential flight performance, which they did not fully utilize during foraging flights.

## Teaser

**Summary:** Accelerometery analysis of pied flycatcher flight behaviours suggests that males and females invest equally in parental care, but that this comes at a reduced cost to males because of their higher flight performance potential.

## INTRODUCTION

Bird flight behaviours have been studied for many years, including detailed examinations of flight kinematics (Krishnan et al., 2022; Hedenström and Møller, 1992), aerodynamics (e.g. Muijres et al., 2012a; Alerstam et al., 2007; Pennycuick et al., 2013) and general aspects such as migration flight strategies (e.g. Mitchell et al., 2015; Jiguet et al., 2019; Hedenström, 2024). Despite this, still relatively little is known about the fundamental aspects of what is required from a bird in terms of investment in flight during day-to-day routine transport and foraging flights. In the daily life of a songbird, foraging occupies a large portion of the time budget. To forage, the bird must move through its habitat, which is done predominantly by flying. The daily energy requirements vary throughout the year (Evans et al., 1994). One of the most demanding periods of the annual cycle is the phase of the breeding period when parent birds provide food for their growing young. Because of the logistical challenges of studying detailed behaviours in the field, particularly little is known about the foraging behaviour of especially small forest songbirds in the wild.

In this study, we investigated the flight activity and effort of both male and female pied flycatchers (*Ficedula hypoleuca*) during the chick rearing period. Pied flycatchers have an active foraging behaviour, feeding on live insect prey, often captured mid-air (Böhm and Kalko, 2009). This foraging behaviour may involve high performance flights with high demands for agility and endurance, making these birds ideal for studying flight activity patterns. A lot is known about pied flycatcher kinematics and aerodynamics from previous wind tunnel and lab studies, which is relevant when attempting to understand and explain their flight behaviour and activity in the wild. This includes detailed knowledge about the wing and tail kinematics of both steadily flying and manoeuvring flycatchers, the underlying fluid mechanics, and the aerodynamic cost and efficiency of flight (Muijres et al., 2012a,b; Johansson et al., 2018; Tomotani and Muijres, 2019).

Furthermore, as nest box breeders, pied flycatchers are an excellent study subject for field studies. Nest box breeders are easy to monitor, as is often done in large multi-year projects (e.g. Lamers et al., 2023). Here, the breeding behaviour of many pied flycatcher pairs in a population is monitored in parallel, by recording hatching date, brood size, and identity of individuals and breeding pairs through metal rings. This makes it possible to relate experimental measurements to breeding stage, allowing for subsequent comparisons between individuals. Such studies enable researchers to discern how males and females within a breeding pair differentially invest in rearing the young (Davies, 1986).

Traditionally, such field studies relied on direct human observations (e.g. Alatalo et al., 1982; Alatalo and Lundberg, 1984). However, new technologies have made it possible to study birds remotely through, for example, radar (Alerstam et al., 2007), radio telemetry (Taylor et al., 2017) and miniature logging devices (e.g. Ropert-Coudert and Wilson, 2005; Stidsholt et al., 2019). Several behavioural and positional parameters can be recorded by loggers attached to animals with little disturbance to their daily activities. Accelerometers, which record accelerations over time, allow the study of animal activities without the limitation of visibility and observer bias (Brown et al., 2013). Accelerometers can be used to record coarse flight activity patterns of birds over a complete annual cycle (Bäckman et al., 2017; Norevik et al., 2019; Macias-Torres et al., 2022). What these accelerometer studies do not show, however, is activity patterns and detailed flight performance at high temporal resolution. Recent applications using supervised machine learning methods (i.e. where annotated behaviours from direct observation of the tracked individual are necessary for model training) can now identify complex behaviours such as food ingestion in spoonbills (Lok et al., 2023). Therefore, we applied a similar supervised machine learning model to pied flycatchers (Yu et al., 2023) in this field study. In addition, dynamic body accelerations (i.e. the acceleration caused by animal body movement) derived from raw accelerometer measurements are widely used as a proxy for energy expenditure (Wilson et al., 2006; Gleiss et al., 2011; Wilson et al., 2019; Sutton et al., 2023). Therefore, we used VeDBA (vectorial dynamic body acceleration) in this study for flight effort comparisons.

In this study, we aimed to unravel the natural foraging flight behaviour of pied flycatchers when breeding in the wild, and how male and female pied flycatchers differentially invest in chick rearing (Mand et al., 2013). We aimed to do this by monitoring the foraging flight activity of breeding pairs using animal-mounted accelerometers. Game theoretical analysis shows that in monogamous pairs where both parents feed the young, any change in feeding effort by one member of the pair should be countered by a change by the other (Houston and Davies, 1985). With realistic slopes of the reaction curves, the resolution may be an evolutionarily stable strategy where the two parents invest equally in feeding, whereas the total effort by the couple should increase with increasing brood size (Houston and Davies, 1985). We therefore hypothesized that males and females devote a similar amount of time and effort to foraging flights, and that flight activity and effort increase with increasing brood size.

Previous research on both Atlas and Iberian populations of pied flycatchers found that males had relatively longer wing length and lower body mass than females (Potti et al., 2016). These differences in wing and body morphology directly affect flight performance (Hedenström and Møller, 1992), and so we also tested how differences in morphology affected the flight activity and effort of pied flycatchers foraging in the wild. We based this test on a simple flight performance model. The aerodynamic thrust force (**T**) produced by a wing scales linearly with wing area (**T**≈*S*). Furthermore, Newton's second law of motion states that a flying bird accelerates proportionally with the thrust force-to-weight ratio (**A≈T**/*m**g***, where *m* is body mass and ***g*** is acceleration due to gravity). Combining these two mechanisms suggests that the in-flight accelerations scale linearly with the weight-normalized wing surface area, which equals the inverse of wing loading (S*=*S*/*m**g***=1/*N*, where *N* is wing loading). Based on this, we hypothesize that males with relatively lower wing loading have higher in-flight accelerations than females. We tested this hypothesis by quantifying the wing and body morphology of our birds (S*=1/*N*), and testing how our measured in-flight accelerations (VeDBA) correlate with these morphological characteristics.

## MATERIALS AND METHODS

### Miniature accelerometers

**Fig. 1.**
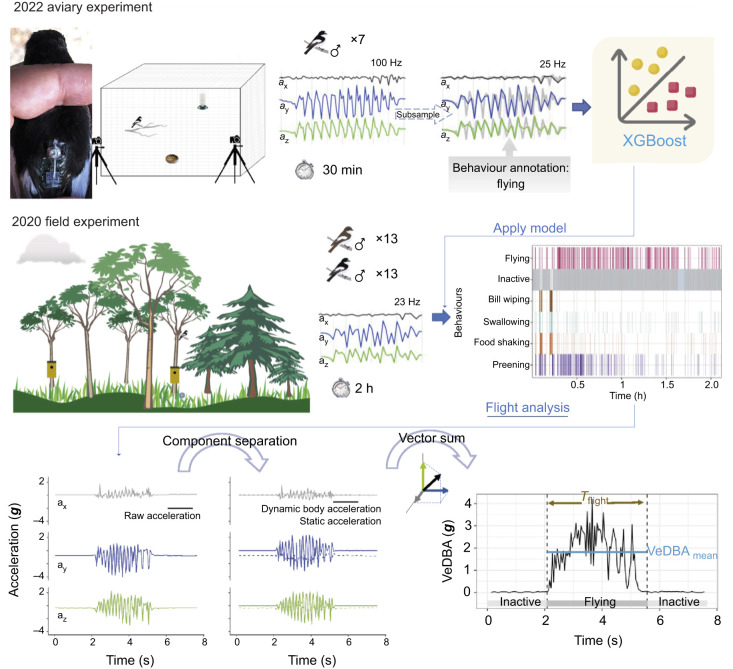
**Diagram of the experimental setup for 2 years.** In 2022, seven male pied flycatchers undertook aviary experiments. Each of them was tagged with an accelerometer logger (the same as in the 2020 experiments – see below). Activities of each bird were recorded by the accelerometer logger as well as two cameras for 30 min. Accelerometer data were subsampled from 100 Hz to 25 Hz to be comparable to the 2020 field experiment sampling frequency. Then, the accelerometer data were annotated to six behaviour types through video records and the annotated dataset was used to train an XGBoost supervised machine learning model (Chen and Guestrin, 2016). In 2020, 13 male and 13 female pied flycatchers were tagged with accelerometer loggers, which recorded accelerometer data at 23 Hz for around 2 h duration when the tagged bird moved freely in their natural environment. The XGBoost model from 2022 was used to predict behaviour types of birds tracked in 2020. VeDBA, vectorial dynamic body acceleration.

The accelerometer device (Fig. 1) used for this study was designed and developed by the Electronics lab at the Department of Biology, Lund University, Sweden. It is a small device (18×9×2 mm, W×L×H) and it weighs 0.7 g. The logger contains an LED light, a light sensor (for activation), a micro-electromechanical system (MEMS) accelerometer, a processor, a zinc-air button cell (A10, 100 mAh capacity) and a non-volatile memory. The accelerometer unit was set to record three-axis acceleration vector [**A**=(*a_x_*,*a_y_*,*a_z_*) in ***g***-force where 1 ***g*** equals 9.81 m s^−2^, with the *x*-axis in the lateral direction, the *y*-axis longitudinal and the *z*-axis vertical], at a sampling frequency of 23 Hz for the field measurements and 100 Hz for the aviary measurements. The measurement range was set to ±8 ***g*** with 8-bit output resolution for each axis, which gives 256 levels and thereby 0.063 ***g*** resolution. The loggers were programmed to include a 30 min delay from activation until the start of data collection to allow birds to resume activities after being handled. Once sampling had started, the device recorded the birds' accelerations continuously for approximately 2 h in the 23 Hz configuration and 30 min in the 100 Hz configuration, limited by memory size, which could store approximately 175,000 individual 3-axis recordings.

### Field measurements

Field measurements were performed in June at Vombs fure, Lund, Sweden, a forest habitat where around 400 monitored nest boxes are available for passerines to breed in (coordinates in decimal degrees: 55.66301, 13.55550; Fig. 1). We successfully recorded activity for 26 pied flycatchers, *Ficedula hypoleuca* (Pallas 1764), in total, 13 males and 13 females. The birds were caught at their nest boxes using efficient and humane methods, primarily through spring-loaded aluminium trap doors at the entrance hole. The handling of the adult birds for measuring, attaching and removing the loggers took a few minutes.

The accelerometers were placed over the synsacrum of the adult flycatchers using a leg-loop harness (Rappole and Tipton, 1991), made from 0.7 mm elastic wire. The battery, albeit very small and lightweight, is the heaviest component of the logger, so to balance the weight of the accelerometer it was mounted with the battery end towards the front of the bird. This way, the heaviest part of the logger is close to the centre of mass of the bird, which should minimize any detrimental pitch moment and its influence on flight performance.

To record wing morphology, we took photos of the wings using a ruler as a reference scale in the photo, where one wing of the bird was spread manually. The weight of the bird with the logger was recorded. After we had deployed the logger on the bird and measurements had been taken, we immediately released the bird close to its nest box.

The logger was typically retrieved later the same day (*n*=17) by catching the individual again (seven loggers were retrieved the following day and two were retrieved 2 days after, because of a failure to re-capture on the same day). Data were later downloaded, after which the logger could be reused. In total, we had 12 loggers at our disposal. The average body mass of our flycatchers was 12.5 g, so the logger weight was approximately 5% of this. The capture and experimental protocols were approved by Malmö-Lund University Animal Ethics Committee (Permit nos 5.8.18-05926/2019 and 5.8.18-05284/2022).

### Aviary measurements

To be able to extract detailed behaviours from the acceleration data, we applied machine learning techniques. In order to apply a supervised machine learning method that require annotating behaviour types to raw accelerometer data, we carried out aviary behaviour observations of seven pied flycatchers in June 2022 (Fig. 1). Details of the aviary experiment can be found in Yu et al. (2023).

### Machine learning

The details of aviary-based behaviour annotation, and machine learning model training and validation can be found in Yu et al. (2023). Importantly, here, we removed the category ‘other’, as it only constituted 1% of the flycatchers' time budget and is not relevant in this study. Therefore, six behaviour categories – flying, inactive (also includes behaviours when the birds were perching, e.g. vigilance or searching for food while perching), food shaking, preening, swallowing and bill wiping – were classified by the XGBoost machine learning method (Chen and Guestrin, 2016). As there was a mismatch between sampling frequency of aviary-based experiments (100 Hz) and the field study (23 Hz), we used subsamples of aviary-based data by taking every fourth data point (tri-axial accelerometer) from the original dataset. Each sample window for behaviour classification contained 16 datapoints, each of approximately 0.7 s duration. This duration was long enough to cover multiple cycles of a specific behaviour (e.g. 14 cycles of wing beats if the bird flies at 20 Hz wingbeat frequency). In addition, the period duration was relatively short compared with that in other studies (Yu et al., 2022; Aulsebrook et al., 2024), to minimize the likelihood of including multiple behaviours in one period.

The behaviour classification model trained on aviary-based data was then applied to the field data (Fig. 1). As we were primarily interested in flight behaviour, we converted all 0.7 s sample window classifications into flying or non-flying. Next, to reduce misclassification, we changed all single ‘non-flying’ sample windows between two ‘flying’ ones into ‘flying’, as we assumed that such a short inactive phase within a flight bout should only be considered as a brief pause and hence should be viewed as part of the same continuous flight. We then divided the filtered flight sequences into a series of flying and non-flying segments, whereby each segment was given its own start time and duration value.

### Data processing and analysis

#### Quantifying flight activity and flight effort

Based on the flights identified by the XGBoost machine learning model, we derived several flight attributes (Fig. 1). These data were used to estimate both the flight activity and flight effort. We quantified flight activity using a combination of two parameters: (1) the flight proportion (*R*_flight_) quantified the relative time spent flying, and (2) mean flight duration (*T*_flight_) was the average duration of all recorded flight bouts. We estimated *R*_flight_ as the total duration of flights divided by the duration of the full recoding sequence. *T*_flight_ was defined as the total duration of flights divided by the total number of flights.

We estimated flight effort based on the recorded in-flight accelerations, as quantified using VeDBA. The calculation of VeDBA followed the method described by Qasem et al. (2012). We first converted the raw accelerometer data recorded by the loggers (**A**) into dynamic body acceleration (DBA) values. We did so by first smoothing accelerations along each axis to derive the static acceleration (**A**_static_) using a running mean over a time duration of 0.22 s (i.e. 5 samples at a 23 Hz sample rate), and then subtracting the static acceleration from the raw data [DBA=(**A**−**A**_static_)]. Finally, the VeDBA values were calculated as:
(1)

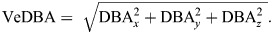
From this, we then calculated the flight effort per flight segment as the mean VeDBA value during that segment (VeDBA_mean_). Finally, we estimated the average flight effort per individual as the average VeDBA_mean_ for all flights performed by that individual (VeDBA_mean,bird_). It is worth pointing out that flight effort in this study does not represent energy expenditure and the ‘effort’ term in this study indicates VeDBA.

#### Wing and body morphology

We determined the mass *m* (kg) of all birds by weighing them using a Pesola spring scale, and we characterized the wing morphology using photos of the wings captured at the time of logger attachment. From these photos, we determined single wing area (m^2^) and semi-span (m) using ImageJ (National Institutes of Health, Bethesda, MD, USA), and following the procedure established by Pennycuick (1989). We then doubled these measures to get the complete wing area *S* and the full wingspan *b*. Mean chord (m) was then calculated as 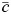
=*S*/*b*, aspect ratio as AR=*b*^2^/*S* and wing loading as *N*=*m****g***/*S* (N m^−2^), where ***g*** is gravitational acceleration (9.81 m s^−2^).

#### Statistical analyses

All statistical analyses were carried out using RStudio (4.1.3). The primary aim of this study was to unravel how male and female pied flycatchers differentially invest in chick rearing by varying their flight activity and flight effort. Hereby, we hypothesized that males and females devote similar amounts of time to flight activity (i.e. mainly foraging flights), and that flight activity increases with increasing brood size. To test these hypotheses, we used a series of statistical tests to unravel how our flight activity and effort metrics (*R*_flight_, *T*_flight_ and VeDBA_mean,bird_) varied between sexes, with brood size and throughout the time of day.

First, we used independent samples *t*-tests to test how flight activity and effort differed between sexes on all tagged birds. We used paired samples *t*-test to test how flight activity and effort differed between sexes for six breeding pairs, i.e. 12 individuals out of the 26 tagged birds. Second, we tested for the effect of brood size on flight activity and effort using two statistical approaches. We used a series of one-way ANOVA to examine the relationship between brood size and the flight activity and effort parameters. Finally, we evaluated whether the time of day influenced the flight activity and effort of our foraging pied flycatchers. For each individual, 1 h after accelerometer device monitoring (i.e. around half of the total accelerometer working time) was selected as the tagging time parameter. We then used a generalized additive model to evaluate the relationship between flight proportion and tagging time.

Flight effort might also vary with flight duration, where for example in-flight insect catching would result in short flights with high activity, and long commuting flights would require lower effort. We therefore also tested for a correlation between VeDBA_mean_ and flight duration (*T*_flight_), for all flights from all tagged individuals. For this, we used maximum likelihood estimation between the parameters with Weibull distribution, assuming the shape and scale of the distribution are influenced by flight duration.

Male pied flycatchers tend to have a relatively lower wing loading (*N*) than females, which might affect their flight performance. Based on this, we hypothesized that these males have better flight performance than females, and as a result perform more rapid foraging flight manoeuvres when foraging in the wild. We tested this hypothesis using two sets of statistical tests. First, we used a set of independent samples *t*-tests to test how wing and body morphology differed between sexes. Second, we used a linear Pearson correlation test on the relationship between the flight effort metric VeDBA_mean,bird_ and weight-normalized wing surface area (S*=1/*N*). Finally, we used this correlation to estimate the difference in flight performance between male and female pied flycatchers and relate this to the foraging flight effort that they exhibited in our study.

## RESULTS

### Machine learning model for behavioural identification

The classification performance by the XGBoost model can be found in the confusion matrix (Fig. S1). Flying and perching had the best overall precision and recalls. For food consumption-related behaviours, swallowing and food shaking were sometimes misclassified between each other. Bill wiping had the lowest recall rate (38.46%) and was often misclassified as food shaking.

### Flight activity and effort of foraging pied flycatchers

**Fig. 2.**
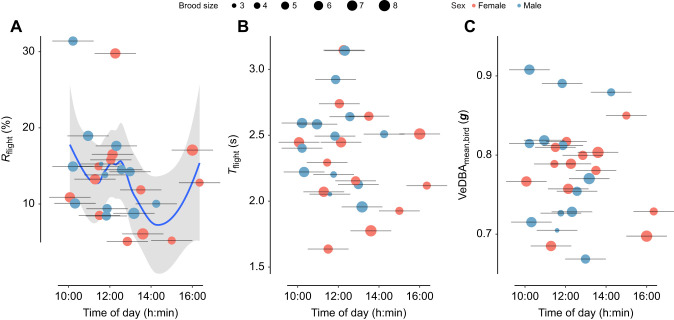
**Flight proportion, duration and effort in relation to the time of day of sampling (tagging time).** (A) Flight proportion *R*_flight_, (B) flight duration *T*_flight_ and (C) flight effort VeDBA_mean,bird_. The horizontal bars show the sampling period for each individual; the corresponding data points show the mean tagging time per sampling used; data points are colour coded in red and blue for females and males, respectively. In A, the blue curve fitted on all data points shows the Loess fit to the data, and the grey shading represents the confidence interval of the curve.

Raw accelerometer data from 26 tagged individuals were classified into six behaviour types by the XGBoost machine learning model, and then converted into flight and non-flight segments. For each flight segment, we estimated the corresponding activity and effort metrics. An overview of flight proportion, flight duration and flight effort in relation to time of day is shown in Fig. 2.

### Flight activity: flight proportion and duration

**Fig. 3.**
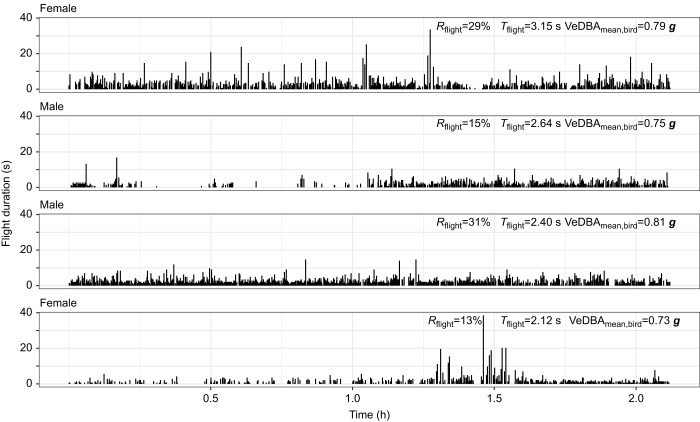
**Time allocation and duration of all identified flight segments of four individuals during ∼2** **h accelerometer recordings.** Flight activity was detected by the XGBoost machine learning model. Each bar shows the start and duration of a single flight segment (on the abscissa and ordinate, respectively). The flight proportion *R*_flight_, mean flight duration *T*_flight_ and flight effort VeDBA_mean,bird_ of each individual are annotated in the corresponding panel.

We quantified flight activity using both flight proportion and flight duration (Fig. 3). All flights from the posted processed sequences showed large variation of flight proportion and duration between individuals. The pied flycatchers had a flight proportion that averaged 13.66% of time (*n*=26, s.d.=6.28%, range=5.08–31.39%). The average number of flights per hour was 199 (*n*=26, s.d.=92, range=52–549). The mean flight duration averaged 2.38 s (*n*=26, s.d.=0.38 s, range=1.64–3.15 s). Short flights with duration less than 10 s accounted for 99.29% of all the flights identified from the pied flycatchers, among which very short flights that lasted less than 1 s were the most frequent (Fig. S2).

### Flight effort: mean VeDBA during flights

**Fig. 4.**
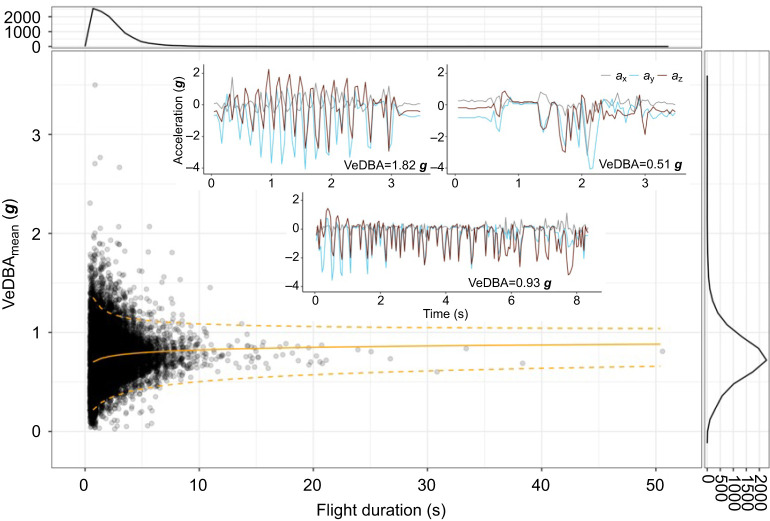
**Flight effort as expressed by VeDBA_mean_ during all 10,909 identified flight segments of the 26 pied flycatchers versus flight duration.** Each dot depicts the mean VeDBA and the corresponding flight duration for a specific flight segment (thus, we show in total 10,909 dots). The orange line was fitted by Weibull distributions, with scale and shape parameters influenced by log-transformed flight duration. The solid orange line shows the median of the fitted distributions, whereas the bottom and top dashed orange lines show the 5% and 95% quantiles of the fitted distribution, respectively. The insets display raw accelerometer data of three flights with high (left), low (right) and near-average (bottom) VeDBA values. In the low VeDBA plot (i.e. VeDBA=0.51 g), raw accelerometer data of all three axes remained close to 0 ***g*** around time 1 s. This is due to the bounding flight pattern, where the flycatcher folds its wings and performs a ballistic manoeuvre, which was confirmed in our 2022 aviary experiment (Fig. S5). The frequency plots (top and right) show the number of occurrences of different flight durations and mean VeDBA values, respectively.

For all the identified flight segments, we estimated the mean acceleration per segment (VeDBA_mean_) as a metric for flight effort (Fig. 4). Short flights had larger variation of VeDBA_mean_, whereas longer flights generally converged to an average VeDBA_mean_ of approximately 0.76 ***g***, which was supported by the fitted distributions in Fig. 4. Short flights with high VeDBA_mean_ values were most likely flights where the bird flew up with continuous strenuous wing flaps or was performing rapid manoeuvres. Slow flights with low mean VeDBA values were possibly associated with descending flights. The long flights with average VeDBA_mean_ were most likely commuting bounding flights at an approximately constant flight speed (Rayner, 1985).

### Differences in flight activity and effort between sexes

Based on the flight metrics per segment (*n*=10,909 flights), we estimated flight activity and effort per individual bird (*n*=26 birds), and compared how these differed between sexes (*n*=13 males; *n*=13 females).

#### Flight activity: flight proportion and duration

**Fig. 5.**
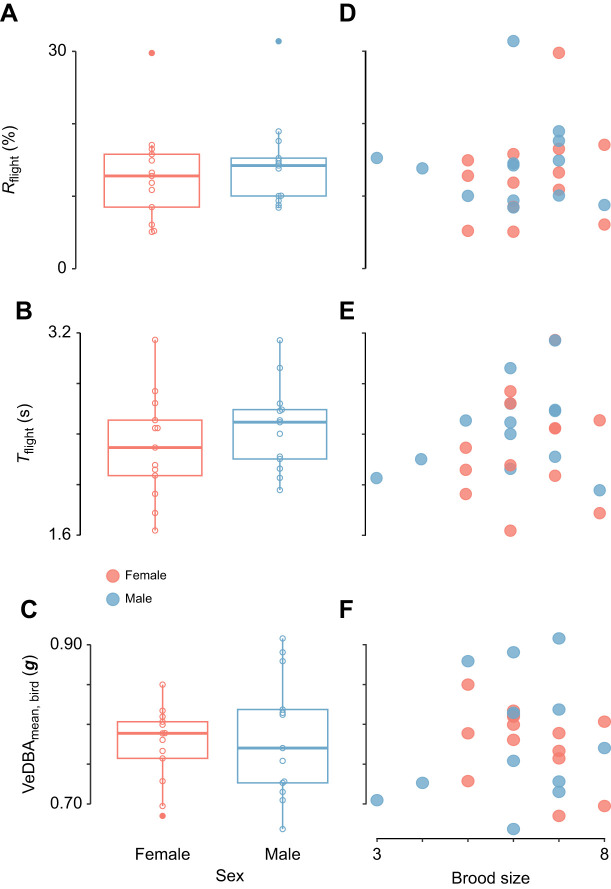
**Flight activity and effort of foraging female and male pied flycatchers (left) and versus the brood size for all studied birds (right).** All data for females (*n*=13) and males (*n*=13) are in red and blue, respectively. The flight activity parameters include flight proportion *R*_flight_ (A,D) and mean flight duration per flight segment *T*_flight_ (B,E); flight effort VeDBA_mean,bird_ was estimated based on the mean in-flight accelerations per bird (C,F). Separate data points show the values per individual, and box plots show median, 25th and 75th percentiles, fences and outliers (filled symbols).

Flight activity did not differ significantly between the sexes (Fig. 5A,B). Specifically, flight proportion (*R*_flight_) showed no significant difference between males and females (Fig. 5A; independent *t*-test on the arcsine square root transformed data: d.f.=23.38, *t*=−0.73, *P*=0.47). Females flew 13±7% (*n*=13) of the time, and for males this was 14±6% (*n*=13). Time spent in behaviours other than flight (i.e. the other five behaviour types) of foraging female and male pied flycatchers is summarized in Fig. S3. Also, flight duration (*T*_flight_) did not differ significantly between males and females (Fig. 5B; independent *t*-test: d.f.=23.14, *t*=−1.00, *P*=0.33). For females, flight segments had a duration of *T*_flight_=2.3±0.4 s (*n*=13) and for males this was *T*_flight_=2.5±0.3 s (*n*=13).

Finally, we performed paired *t*-tests on the flight activity metrics among six pairs to test whether flight activity of the male and female in a single pair differed from each other. For this too, no significant difference was found between the sexes in either flight proportion (arcsine transformed paired *t*-test: d.f.=5, *t*=−0.83, *P*=0.44) or mean flight duration (paired *t*-test: d.f.=5, *t*=−2.2, *P*=0.08).

#### Flight effort: mean in-flight VeDBA per bird

Flight effort as quantified using mean VeDBA did not differ significantly between males and females (Fig. 5C; independent *t*-test: d.f.=20.02, *t*=0.36, *P*=0.72). Females had an in-flight mean VeDBA of 0.77±0.05 ***g*** (*n*=13), and for males this was VeDBA_mean,bird_=0.78±0.08 ***g*** (*n*=13). Paired *t*-tests among six pairs on VeDBA_mean,bird_ also showed no significant difference (d.f.=5, *t*=−0.42, *P*=0.69).

### Effect of brood size on flight activity and effort

We tested for a possible relationship between brood size (range 3–8 chicks) and flight activity and effort.

#### Flight activity: flight proportion and duration

The flight activity metrics flight proportion and flight duration per flight segment did not depend on brood size (one-way ANOVA on arcsine transformed *R*_flight_; *F*=0.72, *P*=0.62, Fig. 5D; one-way ANOVA on *T*_flight_: *F*=1.24, *P*=0.33, Fig. 5E).

#### Flight effort: mean in-flight VeDBA per bird

Flight effort VeDBA_mean,bird_ did not depend on brood size (one-way ANOVA on VeDBA_mean,bird_: *F*=0.81, *P*=0.56).

### Variation in flight activity and effort

Finally, we tested how flight activity and effort varied during a day of foraging, by correlating the average flight proportion per individual with time of day (Fig. 2).

#### Flight activity: flight proportion and duration

From the generalized additive model test, the relationship between flight proportion and time of day was not significant (*F*=2.08, *P*=0.16). However, the fit result shows that foraging flight activity was highest in the morning, when birds flew approximately 15% of the time. Flight activity dipped in early afternoon to 8%, and then increased again towards the late afternoon. The relationship between flight duration and time of day was not significant (*F*=2.4, *P*=0.055).

#### Flight effort: mean in-flight VeDBA per bird

The relationship between flight effort and time of day was not significant (*F*=0.8, *P*=0.48).

In addition, we tested relationships between other environmental factors (i.e. temperature, wind speed and humidity) and flight proportion, and found no significant relationships (Fig. S4).

### Morphological differences between sexes

**Fig. 6.**
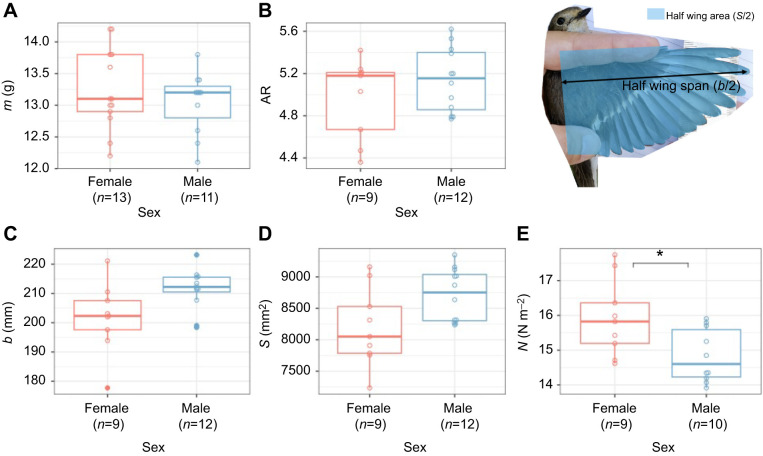
**Morphological differences between the studied female and male pied flycatchers.** All data for females and males are in red and blue, respectively. Measurements included body mass *m* (A), wing aspect ratio AR (B), wing span *b* (C), wing area *S* (D) and wing loading *N* (E). We estimated semi-span (*b*/2) and half the wing surface area (*S*/2) based on digital images of the bird wing (top right). Box plots show median, 25th and 75th percentiles and fences.

None of the primary body and wing morphology parameters differed significantly between males and females (Fig. 6A–D). Body mass did not differ between males and females (males: *m*=13.0±0.49 g; females: *m*=13.2±0.63 g; independent *t*-test, d.f.=21.86, *t*=0.84, *P*=0.41; Fig. 6A). Also, females and males did not have significantly different aspect ratios (AR=4.98±0.38 and AR=5.14±0.3, respectively; independent *t*-test, d.f.=15.05, *t*=−1.07, *P*=0.3; Fig. 6B).

Although also not significant, males appeared to have larger wings (longer wingspan and larger wing area) than females. Males appeared to have a ∼4% larger wing span (males: *b*=211±7 mm; females: *b*=202±12 mm; independent *t*-test, d.f.=12.07, *t*=−2.16, *P*=0.052; Fig. 6C), and a ∼6% larger wing area (males: *S*=8712±418 mm^2^; females: *S*=8196±625 mm^2^; independent *t*-test, d.f.=13.19, *t*=−2.14, *P*=0.051; Fig. 6D).

Despite these non-significant differences in the primary body and wing morphology parameters, the derived wing-loading parameter did differ significantly between males and females (Fig. 6E). Females had on average a ∼7% larger wing loading than males (females: *N*=15.9±1.11 N m^−2^, males: *N*=14.8±0.77 N m^−2^; independent *t*-test, d.f.=14.11, *t*=2.45, *P*=0.028).

### Effect of morphology on flight activity and effort

Wing loading directly affects the ability to produce in-flight acceleration, and thus we hypothesized that these males that tend to have lower wing loading can produce higher in-flight acceleration than females. We tested this by testing how wing loading affects both flight activity and flight effort using linear regressions on the inverse of wing loading, called weight-normalized wing area (S*=1/*N*).

#### Flight activity: flight proportion and duration

We did not find any significant relationship between wing loading and the flight activity metrics flight proportion and flight duration (Pearson correlation test on S* versus *R*_flight_: d.f.=17, *t*=−0.008, *P*=0.99; Pearson correlation test on S* versus *T*_flight_: d.f.=17, *t*=0.33, *P*=0.75).

#### Flight effort: mean in-flight VeDBA per bird

**Fig. 7.**
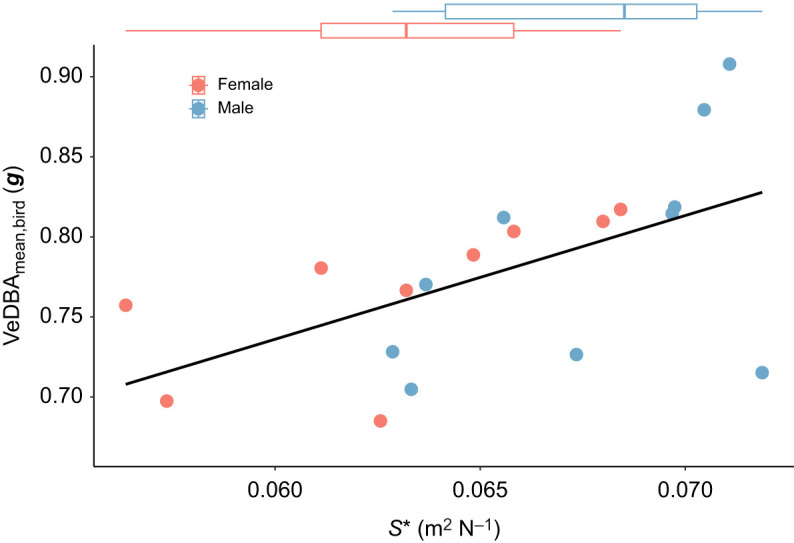
In-flight acceleration versus the weight-normalized wing area S* for all studied birds. All data for females and males are in red and blue, respectively. In-flight acceleration was quantified using the mean acceleration per individual (VeDBA_mean,bird_), and each data point shows the average acceleration (VeDBA_mean,bird_) and weight-normalized wing area (S*) per individual, colour coded by sex. The black line shows the significant linear regression between the acceleration metric and weight-normalized wing area. The box plots at the top show median weight-normalized wing area per sex, and the corresponding 25th and 75th percentiles and fences.

In contrast, mean VeDBA did correlate significantly and positively with weight-normalized wing area (Pearson correlation test on S* versus VeDBA_mean,bird_: *P*=0.011, *R*^2^=0.32; Fig. 7). Thus, among all tested birds, flight effort (as expressed by VeDBA_mean,bird_) scaled linearly and positively with weight-normalized wing area, and thus inversely with wing loading. Because males have on average lower wing loading than females (Fig. 6E), they should also be able to produce high in-flight accelerations, but they did not during the sampled foraging periods (Fig. 5C).

## DISCUSSION

### Foraging flight activity and effort of breeding pied flycatchers

#### Flight activity

The pied flycatchers in our study were actively flying on average 14% of the time during the 2 h window of our observations. At first, this may seem low, considering that these birds are regarded as very active flyers. When not flying or inactive, the birds spent their time preening, feeding themselves and feeding their chicks. It is worth noting that the tagged birds might still be recovering from the disturbance of being captured, handled and tagged.

In a theoretical paper on optimal fuel loads and stopover use during migration, a general proportion of flight and stopover during the complete migration was estimated to be 1:7 for small birds (Hedenström and Alerstam, 1997b), which arises simply as the ratio between power required to fly and the rate of energy accumulation. Interestingly, our flycatchers exhibited a very similar proportion of flying and non-flying, being 1:7.3. This may of course be a coincidence because the condition the birds live under during breeding is vastly different from those during migration, but it is also possible that this stems from a general ratio between time required to fuel to cover a unit of flight time, no matter whether it concerns migratory or foraging flights.

#### Flight duration and number of flights

Flight duration and number of flights of breeding songbirds have rarely been studied previously. Among the tagged pied flycatchers, although there were occasional flights of 30–50 s, most flights were below 10 s in length. The average number of flights per hour was 199. This gives us a picture of how these birds are moving through the habitat, whereby they primarily performed many short flights. With so many flights, it is not likely that the birds only use a sit-and-wait foraging strategy typically associated with these birds. With on average more than three flights per minute, the flycatchers probably used short flights to move through the territory searching for food and only occasionally made aerial forays to catch prey. Indeed, previous studies have shown that prey types that pied flycatcher parents provide to their nestlings include Lepidoptera, Coleoptera, Araneae, Diptera, Hymenoptera, Odonata and Isopoda. Among these, flightless insect larvae seemed to be the dominant food type for nestlings (Lundberg and Alatalo, 2010), which are most likely collected by continuously moving through the territory using series of short flights.

The many short flights have a potential implication for the energy expenditure of the flycatchers. Every time the bird takes off and lands, it accelerates from a stationary position, which takes a lot of energy, relative to steady flight (Nudds and Bryant, 2000). During the consecutive landing manoeuvre, the animal needs to produce large wing forces to brake before touch-down, as well as buffer the forces with the legs and feet at touch-down (e.g. Provini et al., 2014). Changing speed like this must frequently come with added energy expenditure as well as requiring skilful manoeuvring while flying in a complex and dense forest environment (cf. Henningsson, 2021).

Among all flights, short flights (i.e. <10 s) had larger variation of flight effort, as expressed by the mean VeDBA per flight. VeDBA values were found to be positively related to energy expenditure of flying birds (e.g. Sutton et al., 2023). Therefore, the large VeDBA values were possibly associated with ascending flights or rapid flight manoeuvres, which both require increased energy expenditure. However, there was also a large number of short flights with low VeDBA values, possibly associated with descending flights or short flights between tree branches. For flights longer than 10 s, mean VeDBA values were closer to the average VeDBA of all flights, indicating an energy-conserving strategy of bounding flights at constant speeds. Our raw accelerometer data confirm the use of such bounding flights during these long flight segments, as the alternating active flapping and wing folding phases can be observed (Fig. S5).

#### Total flight distance per day

Based on flight proportion, we can estimate the total fight duration and distance travelled by the birds during a day of foraging. As the birds flew on average 13% of the 2 h of monitoring, they were in flight for approximately 15 min. We can extrapolate from this to get a rough estimate of the total flight duration and distance in a single day.

The foraging hours of breeding pied flycatchers have been shown previously to be approximately 17 h per day, from approximately 04:00 h in the morning to 21:00 h in the evening (Lundberg and Alatalo, 2010). During this period, the feeding rate was strikingly constant. With a 13% flight percentage, our pied flycatchers were thus actively flying for approximately 2 h and 13 min during the daily 17 h of foraging.

The average flight speed of migrating pied flycatchers has been measured previously to be 9.74 m s^−1^ (Alerstam et al., 2007). It is worth mentioning that 9.74 m s^−1^ was the estimate of migratory flight speed, which might not be representative of foraging flight speed in this study. Foraging flight speed might be faster according to optimization theory (Hedenström and Alerstam, 1997a). But if we assume this migration speed to be roughly equal to the average speed during foraging, then we can estimate the total flight distance during a single day per individual, based on the 2 h flight activity of that bird. For the 26 monitored birds, the total distance travelled during a day of foraging was 84±38 km, ranging from 31 to 192 km.

According to the Swedish ringing recovery data summarized by Ellegren (1993), pied flycatchers have an average migration speed of approximately 60 km day^−1^ in autumn (*n*=19), and the highest migration speed reached 116 km day^−1^. Thus, this back-of-the-envelope calculation tells us that the daily flight distance of foraging flycatchers during breeding is similar to the distance travelled per night of migration. We often view the migration of long-distance migrants as the most impressive feat of their annual cycle, but feeding their young should not be overlooked in terms of flight effort and energy expenditure.

#### Territory size of breeding pied flycatchers

We found that 99% of flights had a duration below 10 s. If we use the previous estimate of flight speed by Alerstam et al. (2007) of 9.74 m s^−1^ and assume that the birds fly out from the nest and back again in two main flights, this means that 99% of the flights were within 100 m radius of the nest. If we use the mean flight duration of 2.38 s from this study, this means that the average radius is around 23 m. Of course, it could also be the case that the birds fly several consecutive 100 m flights away from the nest and back again and then may reach beyond this radius. This is however a rough estimate and merely an indication of habitat use.

From the perspective of conservation biology, this rough estimation indicates how large territories need to be to sustain a breeding pair, although it will vary with the quality of the territory (Bibby and Green, 1980). How does this compare with other species that feed on different types of food – for example, seed eaters? This needs to be addressed in future studies and would provide invaluable information about breeding bird populations to be used when planning nature reserves or other types of habitat protection. However, 19 out of 26 individuals took occasional longer flights over 10 s and 5 individuals had flights longer than 20 s. These longer flights might be explorational for better foraging habitat or, in the case of males, additional mating opportunities (Alatalo et al., 1982; Lamers et al., 2020).

### Breeding performance and flight activity

We hypothesized that flight activity and effort increase with increasing brood size (Houston and Davies, 1985). Our results do not support this hypothesis, as we did not find a significant correlation between the flight activity and effort parameters and brood size. This could partly be due to the rather low spread of the brood sizes, including only brood sizes from three to eight, and with most samples within five to seven chicks. Future studies could test this by experimentally manipulating brood size. Nevertheless, if for argument's sake no difference was genuinely the case, we could speculate that in natural broods, the parents may not work as hard as they can in order to preserve energy (as a result of parent–offspring conflict), which is similar to the case studied by Masman et al. (1989) in kestrels. They found in their study that male kestrels spent a similar proportion of time in flight irrespective of brood size, but their flight-hunting yield (prey captured per hour flight hunting) was positively correlated with brood size, to guarantee their nestlings had enough food (Masman et al., 1989). However, when kestrels met a situation of food shortage, males allocated more time to flight (Masman et al., 1989). In our case, flycatcher parents with larger brood sizes might either work more efficiently to bring more food back to chicks in each visit (Siikamäki et al., 1998) or occupy better territories where the same flight effort results in more prey.

### Differential flight activity and effort between sexes

#### Differences in morphology and flight effort between sexes

Males had a longer wingspan and larger wing area than females, which is consistent with a previous study (de la Hera et al., 2014). While the aspect ratio between the two sexes was similar, males had significantly lower wing loading. Wing loading directly affects the accelerations that a flying bird can produce, as aerodynamic thrust forces scale with wing area, and in-flight accelerations scale with the ratio between thrust and body mass (Pennycuick, 1989). Our data of 19 foraging pied flycatchers (10 males and 9 females combined) confirm that mean in-flight acceleration (VeDBA_mean,bird_) correlate positively with the inverse of wing loading. But surprisingly, despite their lower wing loading, male flycatchers did not exhibit higher VeDBA values during foraging and chick rearing than female birds (Fig. 5C). This suggests that although male pied flycatchers can produce higher in-flight acceleration than females, they do not do so and thus possibly operate at a reduced relative effort. It is worth noting that tail morphology of birds can also influence their flight performance (Thomas, 1997), which is lacking in this study.

#### Division of parental care labour between sexes

As mentioned above, our paired samples *t*-tests using six pairs showed no significant difference in either flight activity or flight effort between sexes within pairs. This result supports our hypothesis and conclusion that there is no difference in parental investment between the sexes, if it comes to time and activity investment. These tests are relevant as, by comparing between the male and female of the same nest, we can be sure that they have the same ‘task at hand’ as they are feeding the same chicks and therefore face the same challenges. However, in some cases the male may leave the nest and his primary female to occupy a new territory and try to mate with a secondary female (Lundberg and Alatalo, 2010). In polygynous pairs, the parental care the male provides might be less than that of the female, especially in secondary nests (Alatalo and Lundberg, 1984; Lamers et al., 2020). There were only two polygynous cases in our dataset, and only the two females (both secondary females of polygamously mated males) in these two broods were caught and deployed with accelerometers. The frequency of polygyny varies a lot in pied flycatchers between different populations (Lundberg and Alatalo, 2010). More data should be collected in future studies from polygynous pairs (including males and primary females) to test the flight proportion (and mean flight duration) difference between polygynous and monogamous males.

Previous studies found sex differences in foraging behaviours in pied flycatchers, such that females forage relatively more in trees or on the ground, whereas males forage relatively more in the air (Alatalo and Alatalo, 1979; Mand et al., 2013). In our study, although there was no significant difference between females and males in flight proportion *R*_flight_, males had a slightly higher mean *R*_flight_ (14%) than females (13%). In this case, males flew on average 36 s more than females per hour, which might indicate more foraging in the air behaviours in males. Furthermore, males had a slightly higher mean flight duration of 0.2 s than females (i.e. male *T*_flight_=2.5 s and female *T*_flight_=2.3 s), which might also link to more foraging in the air in males.

Our data thus support our hypothesis that female and male partners invest a similar amount of flight time in raising offspring, yet the effort invested during these foraging flights differed. Whilst our in-flight acceleration metric (VeDBA_mean,bird_) did not differ between the sexes, the energy needed to produce the same level of flight performance is dependent on the wing loading. As male pied flycatchers have significantly lower wing loading than females, their similar flight effort indicates that they operate at lower accelerations compared with their potential. A limitation in this study is that we did not differentiate different flight modes (e.g. vertical, low speed, horizontal) from accelerometer data. Different flight modes can affect the energetic budget during parental care. Future research might take this into consideration to have better comparisons between sexes.

Several previous studies have suggested that male and female pied flycatchers invest similar effort in chick rearing (Alatalo et al., 1988; Siikamäki et al., 1998), but these studies lacked detailed information about the in-flight activity of foraging flycatchers. In this study, we show that this apparently equal contribution of males and females to parental care is the result of possible unequal investment in effort. Because of their lower wing loading and consequently higher baseline flight performance, male pied flycatchers might achieve the same parental care output as females, but at lower relative effort. This shows that the balance in parental care between male and female songbirds is more intricate and complex than previously thought. The interpretation of relative effort between sexes through VeBDA in this study should not be directly linked to flight power output or energy expenditure, as these metrics have complex relationships with wingbeat frequency and amplitude, and can vary among species (Krishnan et al., 2022).

The classical ecological studies on breeding birds heavily relied on human observations, and therefore lacked information on birds' activities when they were out of sight. The use of accelerometer tags together with field observation can give a more complete understanding of parental investment in chick rearing. Even so, in this study, we were limited by device storage, i.e. only 2 h accelerometer data were collected for each individual. Therefore, the results need to be taken with caution because of the limited sample time. In future work, edge computing (i.e. processing raw sensor data close to the data source, in this case on-board the bird) could potentially extend the sampling period (Yu et al., 2024) and provide more robust results.

## Supplementary Material

10.1242/jexbio.247606_sup1 Supplementary information
