# Supplementary material for: Flight activity and effort of breeding pied flycatchers in the wild, revealed with accelerometers and machine learning
Source: J Exp Biol. 2024 Oct 10;227(19):jeb247606. doi: 10.1242/jeb.247606 (PMC11491815; doi:10.1242/jeb.247606)
Supplement: Supplementary information [file jexbio-227-247606-s1.pdf]

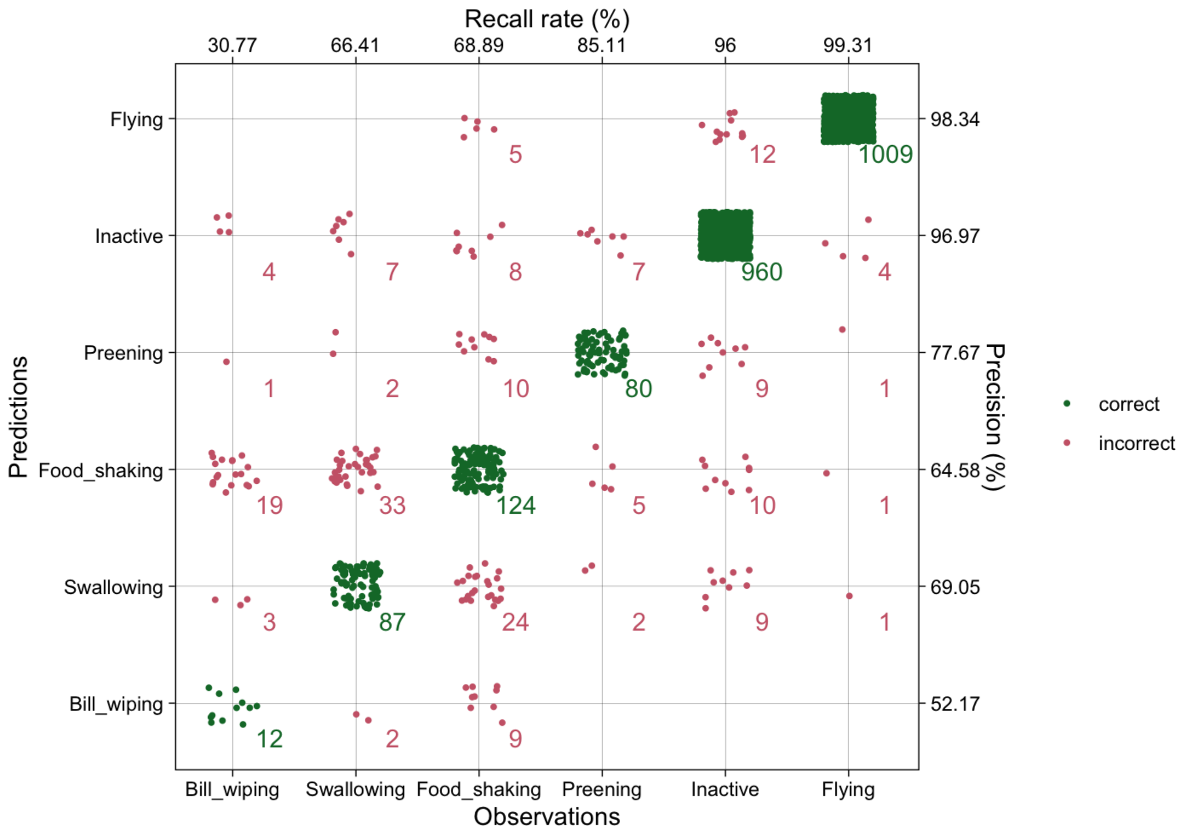

Fig. S1. Predictions by the XGBoost machine learning model versus ground truth behaviour observations. Here, precision rate = TP/(TP+FP) and recall rate = TP/(TP+FN), where TP, FP and FN are the number of true positives, false positives, and false negatives, respectively.

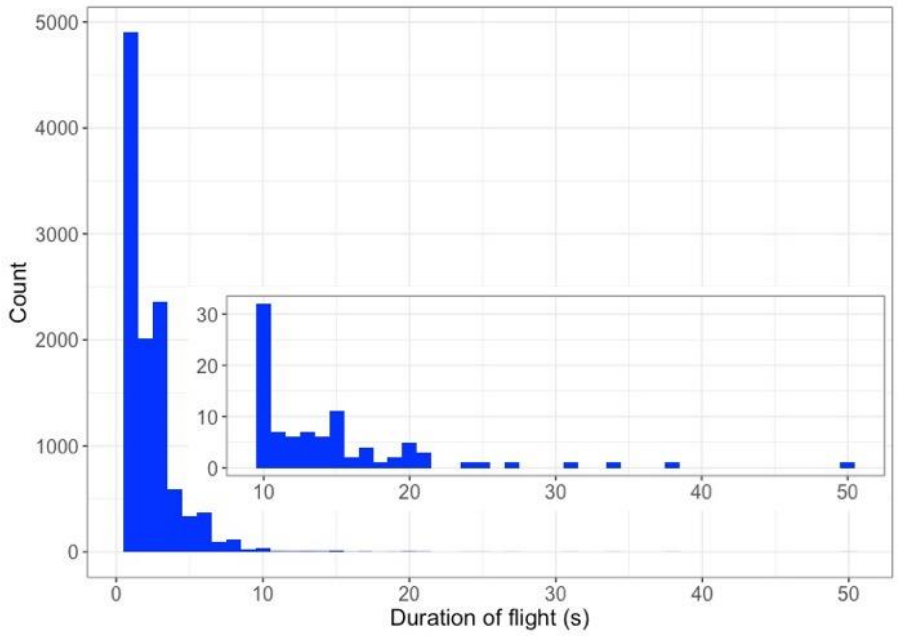

Fig. S2. Histogram of the duration of all 10,909 detected flight segments of the 26 foraging pied flycatchers. The inset shows zoomed in data for the flight with a duration of longer than 10 s.

Journal of Experimental Biology • Supplementary information

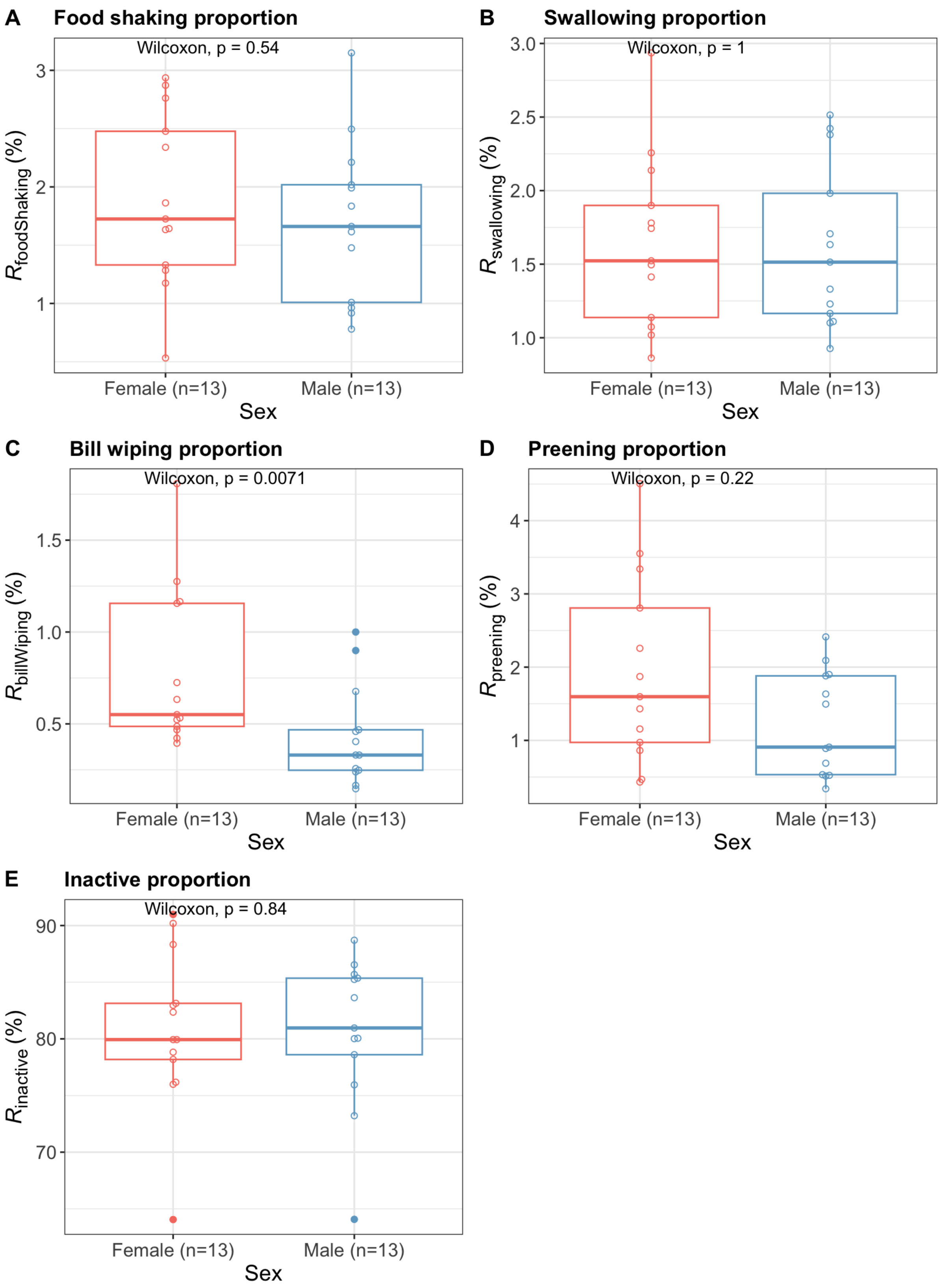

Journal of Experimental Biology • Supplementary information

Fig. S3. Time spent in behaviours other than flights of foraging female and male pied flycatchers, estimated using accelerometer recordings in the field. All data for females (F) and males (M) are in red and blue, respectively. The behaviours include (A) food shaking, (B) swallowing, (C) bill wiping, (D) preening, and (E) inactive. The p values in plots were calculated by Wilcoxon signed rank test between females and males.

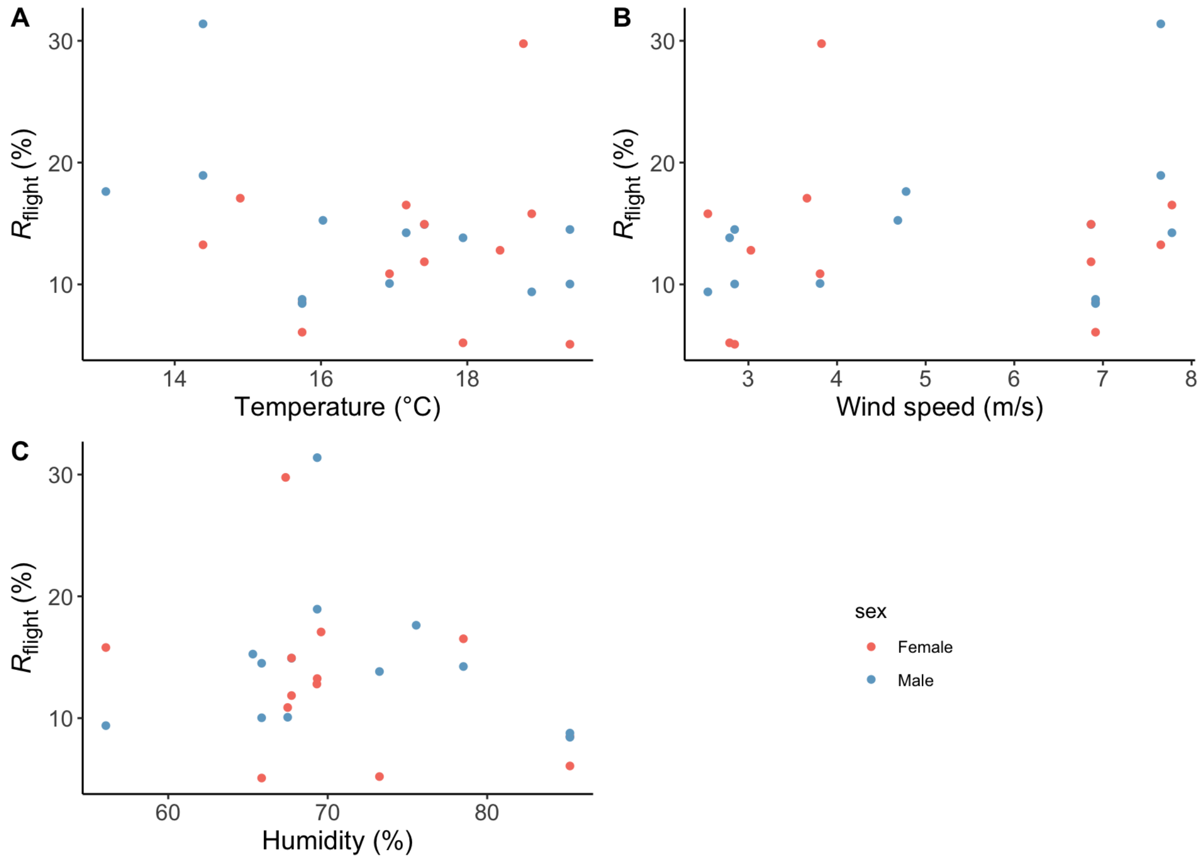

Fig. S4. Relationships between flight proportions R_{flight} of female and male pied flycatchers measured by accelerometer logger and weather parameters (A) temperature, (B) wind speed, and (C) humidity on a daily basis. The weather parameters were from (Boogaard, H., Schubert, J., De Wit, A., Lazebnik, J., Hutjes, R., Van der Grijn, G., (2020): Agrometeorological indicators from 1979 to present derived from reanalysis. Copernicus Climate Change Service (C3S) Climate Data Store (CDS). DOI: 10.24381/cds.6c68c9bb (Accessed on 10-06-2024)). Temperature is air temperature at a height of 2 meters above the surface and we take the mean value of temperatures during daytime. Wind speed is mean wind speed at a height of 10 meters above the surface over the period 00h-24h local time. Humidity is relative humidity at 06h, 09h, 12h. 15h, 18h (local time) at a height of 2 meters above the surface and we take the mean value of these measurement.

Journal of Experimental Biology • Supplementary information

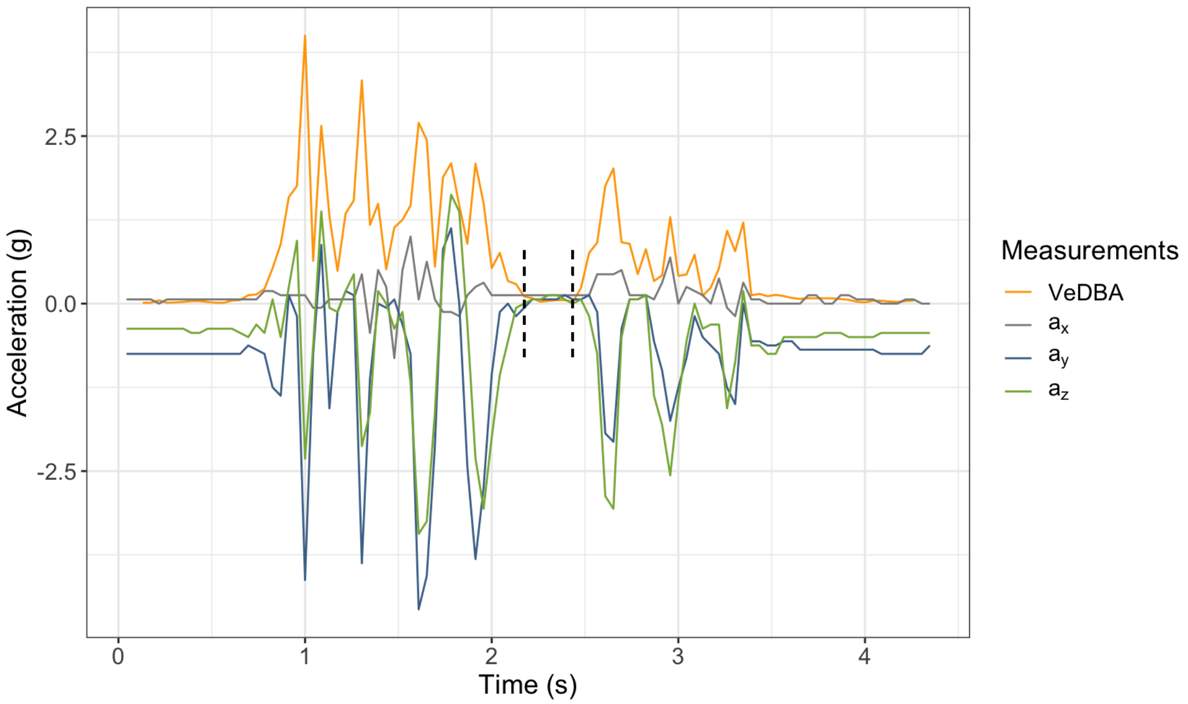

Fig. S5. An example of bounding flight of a pied flycatcher. The period between two vertical dashed lines indicates the wing folding period between active flapping flights. During this period, acceleration recordings along all axes (and VeDBA) are close to zero showing that the animal is performing a ballistic body movement.

Journal of Experimental Biology • Supplementary information
